# ENTPD5 Induces Apoptosis in Lung Cancer Cells via Regulating Caspase 3 Expression

**DOI:** 10.1371/journal.pone.0120046

**Published:** 2015-03-20

**Authors:** Yijun Xue, Lina Wu, Yinan Liu, Yuanyuan Ma, Lijian Zhang, Xuemei Ma, Yue Yang, Jinfeng Chen

**Affiliations:** 1 College of Life Science and Bioengineering, Beijing University of Technology, Beijing, 100022, P.R. China; 2 Key laboratory of Carcinogenesis and Translational Research (Ministry of Education), Central Laboratory, Peking University Cancer Hospital & Institute, Beijing, 100142, P. R. China; 3 Key laboratory of Carcinogenesis and Translational Research (Ministry of Education), Department of Thoracic Surgery II, Peking University Cancer Hospital & Institute, Beijing, 100142, P. R. China; Institute of Biochemistry and Biotechnology, TAIWAN

## Abstract

This study is to investigate the relationship between ectonucleoside triphosphate diphosphohydrolase 5 (ENTPD5) expression and lung cancer clinicopathological factors, and the impact of ENTPD5 on lung cancer cell functions. Lung cancer specimens and matched adjacent normal tissues were obtained from patients without any preoperative radiotherapy or chemotherapy. Knockdown of ETNPD5 expression led to significantly decreased lung cancer cell growth rate, markedly increased apoptosis and the ability to repair, and significantly reduced invasion. Gene chip tests showed that knockdown of ENTPD5 expression caused more Caspase expression. Quantitative real-time polymerase chain reaction showed that the Caspase 3 expression was significantly increased after the knockdown of ENTPD5. In addition, immunohistochemistry showed that the tumor growth marker, proliferating cell nuclear antigen, was significantly reduced in the knockdown model. Tumorigenicity assay and terminal deoxynucleotidyl transferase-mediated dUTP nick-end labeling assay showed that the apoptosis of lung cancer cells was increased in the knockdown model. Our results suggest that ENTPD5 affects lung cancer apoptosis via Caspase 3 pathway, and can be potentially used to monitor prognosis or to guide appropriate therapeutic regimens.

## Introduction

Lung cancer, one of the most common malignant tumors, is the leading cause of cancer-related death worldwide [1]. Non-small-cell lung cancer (NSCLC) approximately accounts for 80% of lung cancer cases [2]. Lung cancer is regarded as a kind of genetic disease in which aberrant endogenous pathogenic gene expression contributes to genomic instability that enhances the motility and invasiveness of cancer cells, leading to the characteristics of invasiveness. Despite successful treatment of the primary malignancy, relapse and subsequent distant metastasis still occur in more than one quarter of postoperative patients [3]. Therefore, postoperative follow-ups should be performed routinely to search for early metastasis to reduce mortality. According to recent research, overexpression of specific genes during carcinogenesis has been detected in several lung cancers, such as epidermal growth factor receptor [4–6], human epidermal growth factor receptor-2 [7], p53 [8] and B-cell lymphoma-2 [9]. Inhibition of apoptosis of tumor cells usually involves many important genes, which may be functionally linked to unlimited cancerous cellular progression such as proliferation, migration and invasion. Eventually, cancerous cells may metastasize to distant organs and threaten lifespan. Genes and proteins that regulate tumor aggressiveness might serve as prognostic markers and/or therapeutic targets of lung cancer. Therefore, it is necessary to develop highly sensitive and specific diagnostic genes/biomarkers to promote accuracy in the early diagnosis of metastasis. By now, several genes have been reported to participate in various pathological processes and significantly influence the aggressiveness of cancerous cells, such as ALK [10], kallikrein-related peptidase 8 gene [11], and RAS [12].

Ectonucleoside triphosphate diphosphohydrolase 5 (ENTPD5) is a kind of enzyme in the endoplasmic reticulum that hydrolyzes UDP to UMP to promote protein N-glycosylation and folding in the endoplasmic reticulum. ENTPD5 protein is distinctive from other NTPDases as it is the only member that is described as a proto-onco protein [13]. ENTPD5 is reported to promote cell proliferation and Warburg effect [13]. Existing evidence confirms that ENTPD5 participates in multiple cellular functional processes and promotes the invasion ability of prostate cancer cells with the help of protein kinase Cδ [14]. Moreover, it is identified that drug-resistance of prostate cancer during platinum-based chemotherapy is related to protein kinase Cδ-mediated stable status of B-cell lymphoma-2 [15]. Previous studies also highlight the importance of ENTPD5 that is associated with tumor formation and cancerous progression of prostate cancer cell lines. These findings demonstrate that down-regulation of ENTPD5 expression negatively influences the capacity for tumor cells to survive in adverse conditions.

There are a lot of reports on the relationship between ENTPD5 and malignant tumor growth, but there is almost no report about the correlation between ENTPD5 and lung cancer. Recently, Curry et al. reported that suppression of ENTPD5 in PTEN null animal model is sufficient to decrease insulin-like growth factor 1 receptor levels and to sensitize bronchiolar tumor cells to serum starvation *in vitro* and to dietary restriction *in vivo* [16]. This study confirms that ENTPD5 might be related to the occurrence of lung cancer in animal experiments.

Considering the deficiency of ENTPD5 research in lung cancer and the important role of ENTPD 5 in the process of tumor development, we designed this study to understand the detailed role of ENTPD5 in lung cancer cell growth and invasion process. In addition, we would like to determine whether ENTPD5 is a promising target in the therapy for lung cancer.

## Materials and methods

### Cells

All lung cancer cell lines, including A549, PC9, H1650, H1975, H1299 Skmes-1, and GLC82, were purchased from the American Type Culture Collection (ATCC, Manassas, VA, USA) and cultured in RPMI-1640 supplemented with 10% fetal bovine serum (Gibco, USA), 100 U/ml penicillin, and l g/ml streptomycin (Invitrogen, Grand Island, NY, USA) in a humidified atmosphere of 5% CO_2_ and 37°C.

The sequences of ENTPD5 siRNA and non-silencing control siRNA were 5’- CCUGGGAUUUGGAUUGAAATT −3’ and 5’- UUUCAAUCCAAAUCCCAGGTT −3’, respectively. The siRNAs were generated by Genepharma (Shanghai, China). The siRNAs were transfected into A549 cells and PC9 cells using Lipofectamine 2000 (Invitrogen, Carlsbad, CA, USA) following the manufacturer’s protocol.

### Patients

Lung cancer specimens (n = 131) and matched adjacent normal tissues were obtained from patients without any preoperative radiotherapy or chemotherapy at Beijing Cancer Hospital from 1999 to 2011. Prior written and informed consent were obtained from all patients and or their families. The study was approved by the Ethics Committee of Peking University. Clinicopathological characteristics of the tumors were defined according to the tumor node metastasis staging system of the Union for International Cancer Control. Clinicopathological factors are shown in Table 1.

**Table 1:** The clinical and pathological characteristics of the patients.

Parameters	No. of cases with respective ENTPD5 staining scores			
	All scores	0	1–3	P value (χ^2^)
**Sex**				
Male	77 (58.8%)	52	25	**0.013**
Female	54 (41.2%)	47	7	
**Age (years)**				
≤ 60	70 (53.4%)	52	18	0.436
> 60	61 (46.6%)	47	14	
**Smoke history**				
Yes	62 (47.3%)	39	23	**0.001**
No	69 (52.7%)	60	9	
**Histological types**				
Squamous cells	21 (16.0%)	14	7	0.582
Adenocarcinoma	93 (71.0%)	72	21	
Others	17 (13.0%)	13	4	
**Cell differentiation**				
Poor	56 (42.7%)	42	14	0.953
Moderate	52 (40.0%)	40	12	
Well	23 (17.6%)	17	6	
**Tumor stages**				
T1	71 (54.2%)	55	16	**0.023**
T2	57 (43.5%)	44	13	
T3	2 (1.5%)	0	2	
T4	1 (0.8%)	0	1	
**Nodal status**				
N0	90 (68.7%)	70	20	0.651
N1	19 (14.5%)	13	6	
N2	22 (16.8%)	16	6	
**Vessel cancer embolus**				
V(-)	102 (77.9%)	79	23	0.526
V(+)	17 (13.0%)	11	6	
Unknown	12 (9.2%)	9	3	
**Tumor node metastasis stages**				
Ia	51 (39.0%)	42	9	0.342
Ib	33 (25.2%)	23	10	
IIa	9 (6.9%)	5	4	
IIb	12 (9.2%)	10	2	
IIIa	23 (17.6%)	16	7	
IIIb	3 (2.3%)	3	0	

### Immunohistochemistry

Primary pulmonary cancer samples were fixed and paraffin-embedded. Sections (5 μm) were routinely processed, and stained with a rabbit monoclonal anti-ENTPD5 antibody (Sigma-Aldrich, Poole, Dorset, UK) at a concentration of 2 μg/ml, followed by the incubation with horseradish peroxidase-conjugated rabbit anti-rabbit secondary antibody (Sigma-Aldrich, Poole, Dorset, UK). Positive staining areas in the entire tissue section were graded by the percentage of positively stained cells: 0, for < 25%; 1, for 25–49%; 2, for 50–74%; and 3, for 75–100%. In this study, 0 was considered negative or moderate staining, while 1, 2 and 3 were considered positive staining.

### Quantitative real-time polymerase chain reaction (qRT-PCR)

Total RNA was extracted using TRIzol reagent (Invitrogen, Carlsbad, CA, USA) following the manufacturer’s protocol. For mature miRNA quantification, a polyA tail was added to total RNA (100 ng) by polyA polymerase (New England Biolabs, Beverly, MA, USA), followed by reverse transcription with oligo-dT adapter primers and Moloney murine leukaemia virus (Invitrogen, USA). cDNAs were synthesized using 2 μg total RNA using the iScript cDNA synthesis kit (Bio-Rad, Hemel Hempstead, UK). Primers for ENTPD5 and GAPDH are listed in Table 2. GAPDH was used as the loading control. qRT-PCR was performed using SYBR Green PCR Master Mix (Toyobo, Osaka, Japan) on Light-Cycler 480 Real-Time PCR System (Roche, USA). The relative amount of miRNAs or genes was normalized to GAPDH. Data were calculated based on 2^-ΔCt^ where ΔCt = Ct (Target)—Ct (Reference). Fold change was calculated using the 2^-ΔΔCt^ method.

**Table 2:** Primer sequences.

GAPDH	Forward	AGGTCGGAGTCAACGGATTTG
	Reverse	GTGATGGCATGGACTGTGGT
ENTPD5	Forward	CATGTGCCCCATTAATGTCAGT
	Reverse	TGCCCGCATCAA ACATAATTC
Caspase 3	Forward	CTCGGTCTGGTACAGATGTCGATG'
	Reverse	GGTTAACCCGGGTAAGAATGTGCA
Caspase7	Forward	TGACAGCCCACTTTAGG
	Reverse	GAACCGTGGAATAGGC

### Western blotting

Proteins were extracted from cells using RIPA buffer containing complete protease inhibitor cocktail (Roche, Mannheim, Germany). Proteins were separated using sodium dodecyl sulfate polyacrylamide gel electrophoresis and transferred to polyvinylidene fluoride membranes. The membranes were then blocked with 5% nonfat dried milk in TBST (15 mM Tris-HCl, pH 7.4, 0.9% NaCl, and 0.05% Tween-20, pH 7.4) followed by blot analysis using specified antibodies: rabbit monoclonal anti-human ENTPD5 antibody (Sigma-Aldrich, Poole, Dorset, UK), and rabbit anti-rabbit secondary antibody (1:1,000). Immunoreactive bands were detected using SuperSignal West Femto Chemiluminescent Substrate. Experiments were performed at least twice with consistent results.

### Cell proliferation assay

Cell proliferation was determined using cell counting kit-8 assay. Cells (2 × 10^3^) were cultured in 96-well culture plates. The cells were resuspended in RPMI-1640 medium containing 10% FBS, and divided into groups before being cultured for 0, 24, or 48 h. The number of viable cells was determined by measuring absorbance at 450 nm using FLUOstar OPTIMA (BMG LAB-TECH, Offenburg, Germany) according to the manufacturer’s instructions. Each experiment was performed in triplicate.

### Flow cytometry

Cells were harvested and assayed for apoptosis using Annexin V-FITC & PI apoptosis detection kit (Imgenex, USA) according to manufacturer’s instruction. Cells were analyzed in FACSCalibur Analyzer (Becton-Dickinson, USA) using the CellQuest Pro software.

### Transwell invasion assay

Transwell invasion assay was performed following the manufacturer’s guidelines. Briefly, 5 × 10^4^ cells cultured in RPMI-1640 medium supplemented with 0.1% FBS were plated into 24-well plates containing RPMI-1640 medium supplemented with 10% FBS as chemoattractant. After 24 h, cells that migrated through and adhered to the other side of the insert were fixed and stained with 0.5% (w/v) crystal violet. Invading cells on the bottom of the filters were imaged using fluorescence microscopy. Five high-power fields were counted per filter to score for invasion. The number of cells was quantified using the ImageJ software.

### Wound healing assay

Cells were cultured to confluence in 6-well dishes before scratching with a 200 μl pipette tip. Debris was removed by extensive washing with phosphate-buffered saline (PBS) and the cells were further incubated for another 24 h or 48 h after injury.

### cDNA microarray analysis

Total RNA was extracted using TRIzol method (Invitrogen, Carlsbad, CA), purified with RNeasy mini kit (Qiagen, Valencia, CA, USA), processed using a GeneChip Expression 3′-Amplification Reagents Kit (Affymetrix, USA), and interrogated with an Affymetrix Primeview Human Gene Expression Array. RNA was further purified using RNeasy mini kit (Qiagen, Valencia, CA, USA) according to Affymetrix introductions. For expression array analysis, total RNA (250 ng) was used to generate biotin-labeled cRNA using the GeneChip Expression 3’-Amplification Reagents Kit (Affymetrix, USA) according to the manufacturer’s protocol. The cRNA was hybridized to Affymetrix Primeview Human Gene Expression Array and scanned using an Affymetrix GeneChip array Scanner 3000 7G.

### Tumorigenicity assay

Six-week-old female Balb/c athymic nude mice (Vitalriver Laboratory Animals, Beijing, China) were subcutaneously injected in the right flank with 2.0 × 10^6^ cells in 0.1 mL PBS. Once tumors were formed, caliper measurements were performed daily and tumor volume (V) was calculated using the formula V = (L×W^2^)/2, where L was the length and W was the width of the tumor. When tumors reached an average volume of 30–69 mm^3^, the mice were randomly divided into three groups (n = 6) for daily intratumoral injection of ENTPD5-siRNA and negative control for 16 days. For each injection, 15 μg miRNA were mixed with 15 μL *in vivo* transfection reagent (Entranster-in vivo, Engreen, China). Growth curves were plotted using average tumor volume within each experimental group at several time points. The tumor volumes of the mice were recorded for 16 days, after which the mice were euthanized. The dissected tumors were collected and prepared for subsequent analyses. All animal experiments were approved by the animal center of the Beijing Cancer Hospital.

### Terminal deoxynucleotidyl transferase-mediated dUTP nick-end labeling (TUNEL) assay

Terminal deoxynucleotidyl transferase-mediated dUTP nick-end labeling (TUNEL) assay kit (in situ Cell Death Detection Kit, Beyotime Institute of Biotechnology, China) was used to assess the number of apoptotic cells. Frozen sections were fixed in 4% paraformaldehyde in pH 7.4 PBS for 1 h at room temperature and permeabilized in PBS with 1.0% Triton X-100 for 2 minutes. The broken DNA ends of dead cells were labelled using TUNEL kit following the manufacturer’s protocol.

### Statistical analysis

Statistical analysis was performed using the SPSS version 18.0 (SPSS Inc., Chicago, IL, USA). Mann-Whitney test was used for the analysis of continuous variables. Chi-square test, Fisher’s exact test or one-way ANOVA test was employed for the analysis of categorical variables. P < 0.05 was considered statistically significant.

## Results

### ENTPD5 scores are correlated to genders, smoke history and tumor stages, with the negative expression of ENTPD5 leading to longer survival time

**Fig 1:**
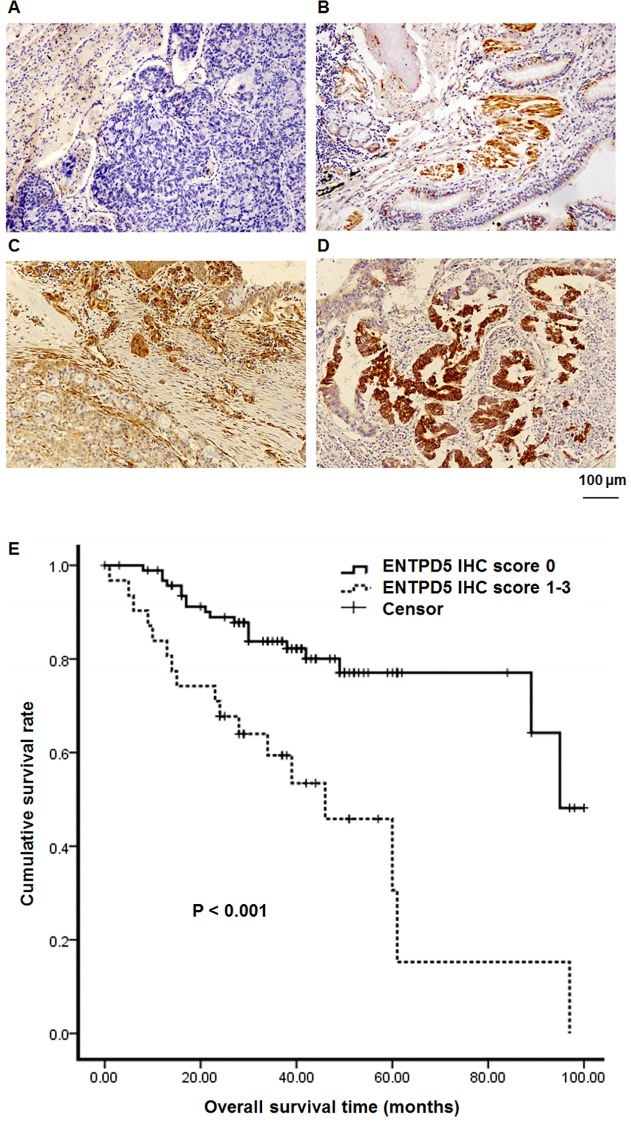
The effect of ENTPD5 scores on pulmonary cancerous tissue specimens and patient survival rate. ENTPD5 staining of lung cancer tissues with **(A)** score 0, **(B)** score 1+, **(C)** score 2+, and **(D)** score 3+. **(E)** Kaplan-Meier analysis of the correlation between overall survival of lung cancer patients and the expression status of ENTPD5 protein (P < 0.001).

To detect ENTPD5 expression in lung cancer tissues, lung cancer cells were stained for immunohistochemical analysis. Cancer cells showed strong and diffuse cytoplasmic staining of ENTPD5 (Fig. 1A-D). High scores of ENTPD5 (1–3) in NSCLC were found in 32 cases (24.4%). Among all the available parameters, sex, smoke history and tumor stages showed significant correlation to ENTPD5 scores (P < 0.05) (Table 1). Univariate analysis of the impact of ENTPD5 expression status on the prognosis showed that longer survival time was significantly correlated with negative expression of ENTPD5 (mean survival time, 81 months vs 45.6 months, P < 0.001). Kaplan-Meier survival curve, based on ENTPD5 expression score, showed that the cumulative survival time for patients with ENTPD5 negative expression (score = 0, n = 96) was significantly longer than that for patients with high levels of ENTPD5 (scores = 1–3, n = 31) (Fig. 1E). These data suggest that ENTPD5 scores are correlated to genders, smoke history and tumor stages, with the negative expression of ENTPD5 leading to longer survival time.

### Inhibition of ENTPD5 expression reduces lung cancer cell growth and increases their apoptosis rate *in vitro*

**Fig 2:**
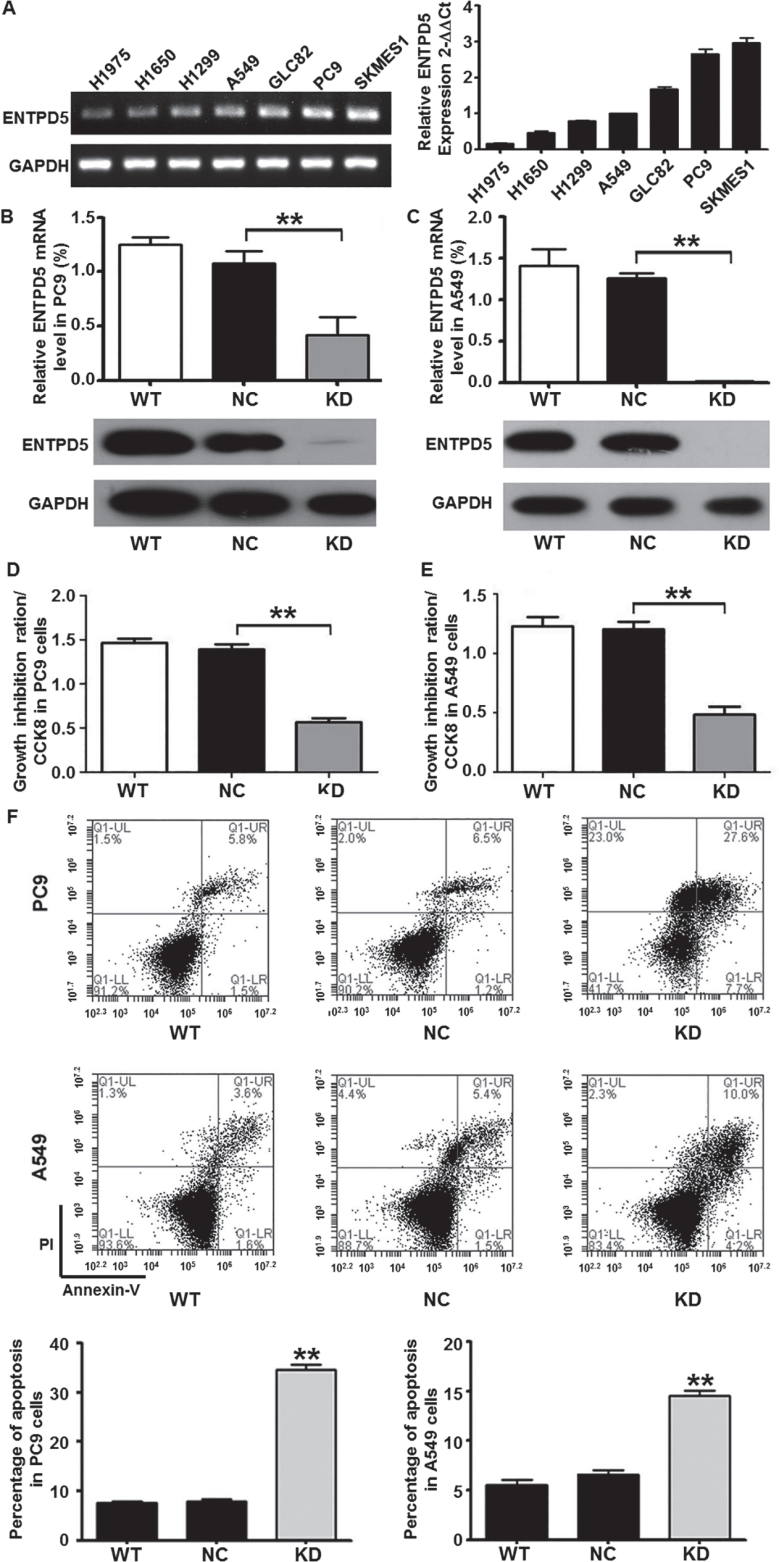
Expression of ENTPD5 in lung cancer cells and its effect on the growth and apoptosis of lung cancer cells. **(A)** Expression of ENTPD5 in different lung cancer cell lines. Relative ENTPD5 expression in **(B)** PC9 and **(C)** A549 cells determined by qRT-PCR and Western blotting. Growth ability of **(D)** PC9 and **(E)** A549 cells after the knockdown of ENTPD5 gene determined by growth assay. **(F)** Apoptosis of PC9 and A549 cells after the knockdown of ENTPD5 gene determined by flow cytometry. Data are means ± SD. Double asterisks indicate values that are significantly different from each other.

To test the effect of ENTPD5 on lung cancer cell growth and apoptosis *in vitro*, we successfully constructed the transient transfection cell models. Evaluation of ENTPD5 expression levels in different lung cancer lines, including H1975, H1650, H1299, A549, GLC82, PC9 and SKMES1, showed that SKMES-1 had the highest relative ENTPD5 expression (Fig. 2A). In addition, knockdown of ENTPD5 induced significantly decreased ENTPD5 expression levels in PC9 and A549 cells (Fig. 2B and C). Furthermore, cell growth assay on PC9 and A549 cells with moderate expression of ENTPD5 showed that knockdown of ENTPD5 significantly reduced the growth of lung cancer cells (Fig. 2D and E) (P < 0.001). Apoptosis analysis on PC9 and A549 cells demonstrated that knockdown of ENTPD5 increased cell apoptosis rate compared with control groups (P < 0.05) (Fig. 2F). These data indicate that inhibition of ENTPD5 expression reduces lung cancer cell growth and increased their apoptosis rate *in vitro*.

### Down-regulation of ENTPD5 expression decreases the invasion ability and motility of lung cancer cells *in vitro*

**Fig 3:**
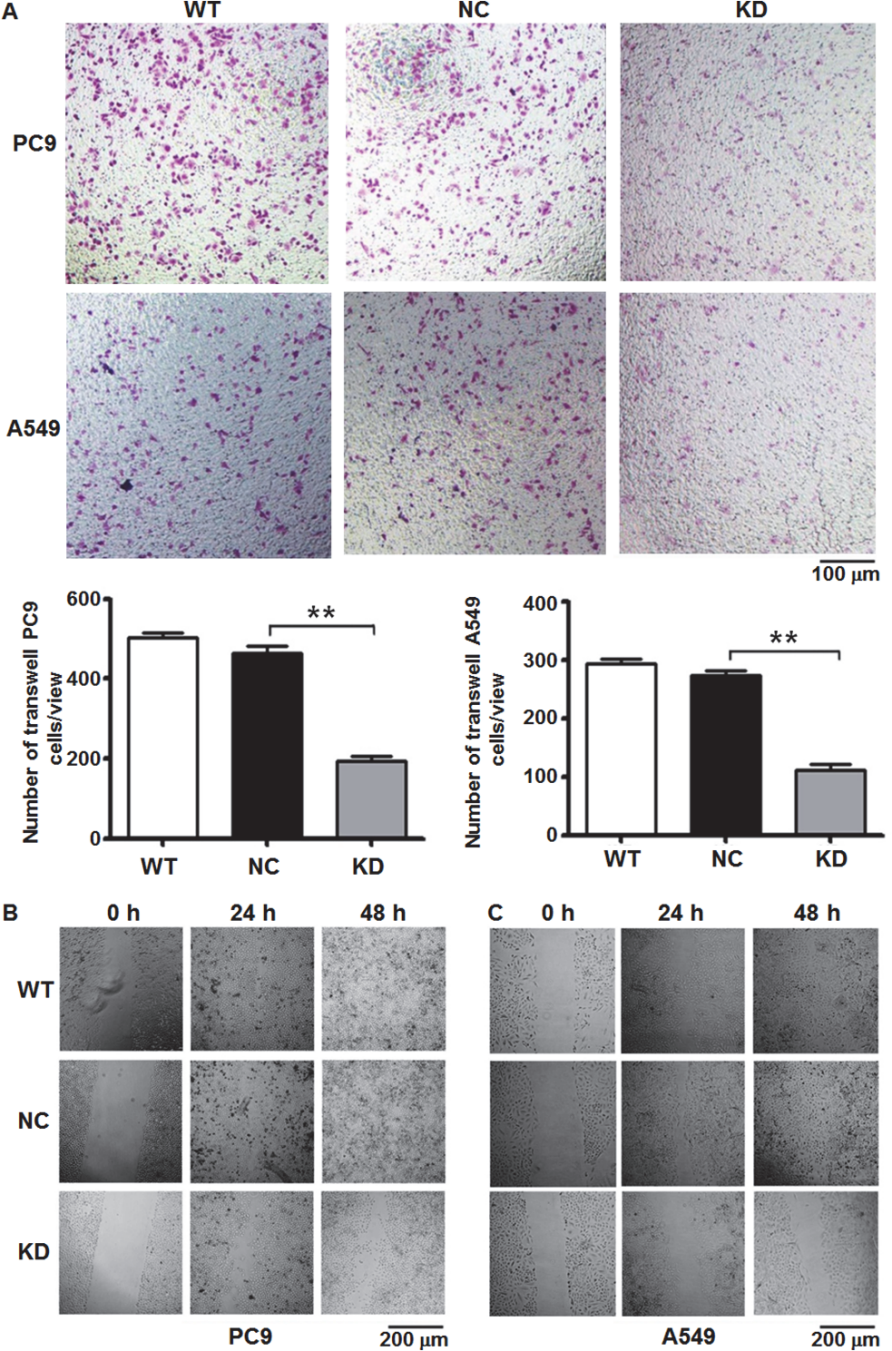
(A) Invasion of PC9 and A549 cells after the knockdown of ENTPD5 gene determined by cell invasion assay. ENTPD5-KD or KD indicates the knockdown of the lung cancer cell lines. Data are means ± SD. Double asterisks indicate values that are significantly different from each other (P < 0.05). **(B)** Wound healing of PC9 and A549 cells after the knockdown of ENTPD5 gene compared with wild-type (WT) and normal control (NC) models at 0, 24 or 48 h. During invasion and wound healing experiments, caspase 3 was added to inhibit cell apoptosis.

To investigate the effect of ENTPD5 on lung cancer cell invasion and motility *in vitro*, transwell invasion assay and wound healing assay were performed. Transwell invasion assay showed that down-regulation of ENTPD5 expression significantly inhibited the invasion ability of PC9 and A549 cells (P = 0.000142 and P = 0.00158, respectively) (Fig. 3A). Wound healing assay demonstrated that down-regulated expression of ENTPD5 dramatically decreased the motility of PC9 and A549 cells from 24 h to the end of the experiments compared with control cells (Fig. 3B and C). These data suggest that down-regulation of ENTPD5 expression decreases the invasion ability and motility of lung cancer cells *in vitro*.

### Caspase 3 might play an important role in the apoptosis of lung cancer cells after the knockdown of ENTPD5

**Fig 4:**
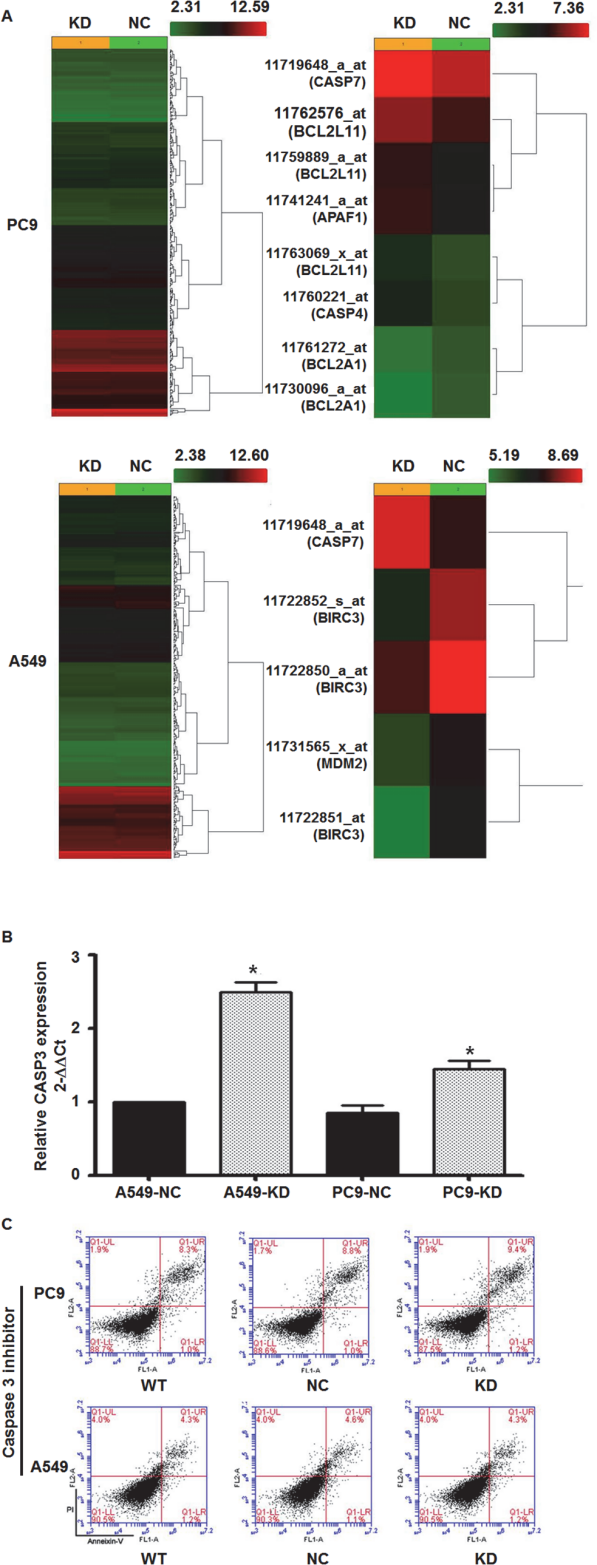
Gene chip test and apoptosis recovery experiments. **(A)** Gene chip test showing pathway analysis that was associated with apoptosis. Caspase gene overexpression was observed in PC9 and A549 cells after the knockdown of ENTPD5. **(B)** qRT-PCR test showing Caspase 3 expression in PC9 and A549 cells after the knockdown of ENTPD5. Data are means ± SD. Asterisks indicate values that are significantly different from NC group (P < 0.05). **(C)** Apoptosis of PC9 and A549 cells after the knockdown of ENTPD5 in the presence of Caspase 3 inhibitor.

To understand the mechanisms of action of proteins related to lung cancer cell apoptosis after the knockdown of ENTPD5, we performed gene chip analysis on PC9 and A549 cells, which was mainly focused on the tests of cell apoptosis pathway. For PC9 cells, knockdown of ENTPD5 increased the expression of some genes by more than 1.5 fold compared with control, including CASP4, APAF1, BCL2L11, CASP7, and BCL2A1. For A549 cells, knockdown of ENTPD5 increased the expression of some genes by at least 2 fold compared with control, including CASP7, MDM2, and BIRC3 (Fig. 4A). Of note, Caspase 7 (CASP7), a member of Caspase 3 subgroup, showed significantly increased expression after ENTPD5 knockdown in both PC9 and A549 cell lines (Fig. 4A). These data suggest that Caspase 3 might play an important role in the apoptosis of lung cancer cells after the knockdown of ENTPD5.

### Caspase 3 inhibitor prevents the increase of apoptosis rate of lung cancer cells induced by the knockdown of ENTPD5

To confirm the importance of Caspase 3 in lung cancer cell apoptosis after the knockdown of ENTPD5, qRT-PCR and flow cytometry were performed. The results of qRT-PCR showed that the relative expression of Caspase 3 mRNA was significantly increased by the knockdown of ENTPD5 in both PC9 and A549 cells (Fig. 4B). In addition, flow cytometry showed that ENTPD5 knockdown failed to induce significant increase of apoptosis rate in PC9 and A549 cells in the presence of Casepase 3 inhibitor (Fig. 4C). These data indicate that Caspase 3 inhibitor prevents the increase of apoptosis rate of lung cancer cells induced by the knockdown of ENTPD5.

### Knockdown of ENTPD5 inhibits the growth and promotes the apoptosis of lung cancer cells *in vivo*

**Fig 5:**
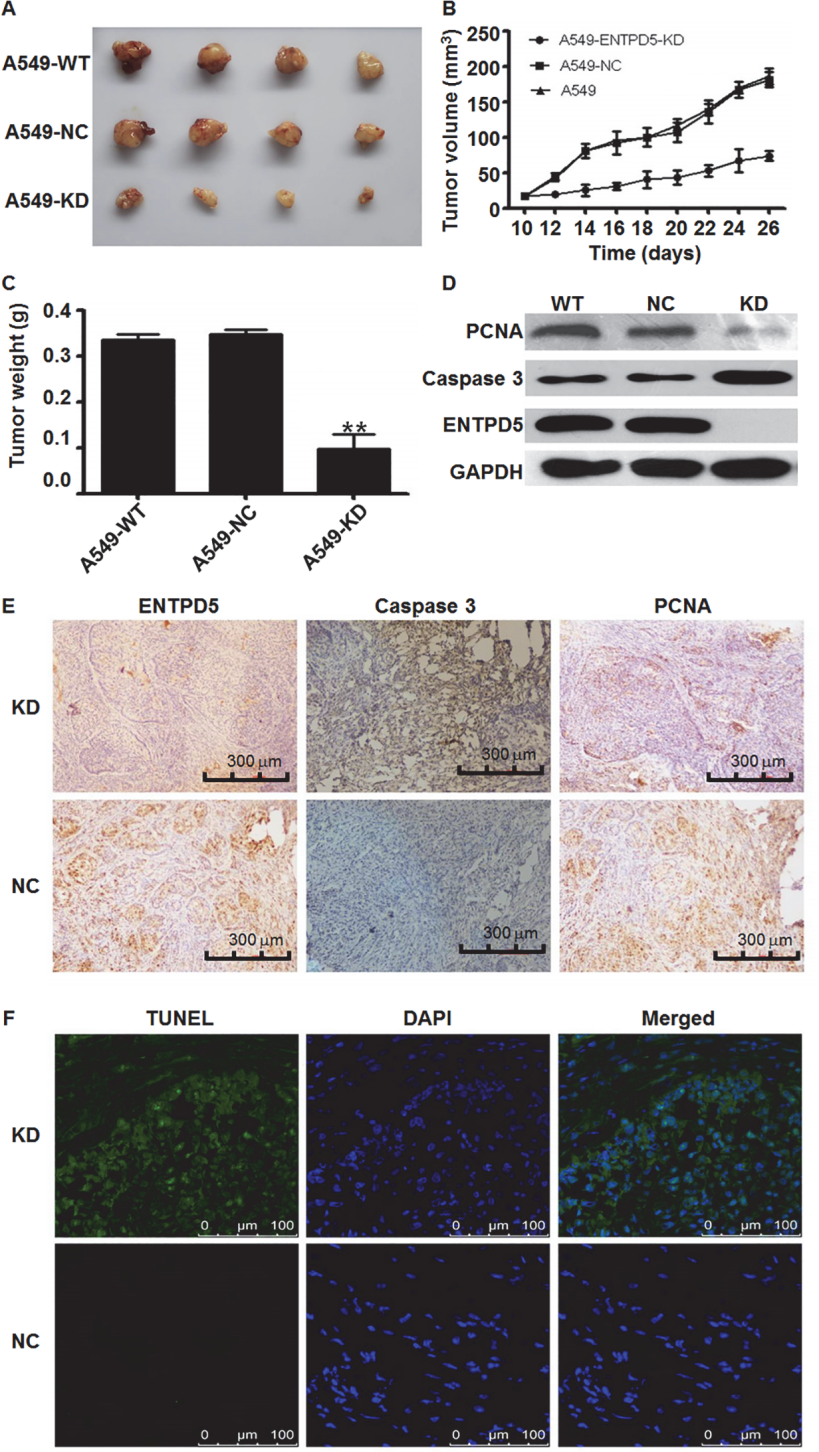
(A) Sizes of the dissected tumors from different groups of A549 cells growing in mice. **(B)** Tumor volume change in different groups of A549 cell injections. **(C)** Tumor weight change in different groups. Data are means ± SD. Double asterisks indicate values that are significantly different from NC group (P < 0.05). **(D)** Proliferating cell nuclear antigen, Caspase 3 and ENTPD5 expression status in different animal model groups determined by Western blotting. **(E)** Immunohistochemistry tests showing ENTPD5, Caspase 3 and proliferating cell nuclear antigen expression status in different animal model groups. **(F)** Apoptosis of tumor tissue cells in animal models after the knockdown of ENTPD5 determined in situ using TUNEL assay.

To validate the effect of ENTPD5 in animal models, tumorigenicity assay, Western blotting assay, immunohistochemistry and TUNEL assay were performed. Tumor growth status in mice injected with A549 cells showed that ENTP5-KD group of mice had smaller sizes of tumor, slower rate of tumor volume growth, and lower final tumor weight than those in WT and NC groups (Fig. 5A-C). Western blotting analysis of tumor tissues showed that the protein expression of proliferating cell nuclear antigen and ENTPD5 was decreased but that of Caspase 3 was increased in ENTPD5-KD group of mice compared with control groups (Fig. 5D).

Immunohistochemical analysis of the expression of proliferating cell nuclear antigen, ENTPD5 and Casepase 3 concurred with the data obtained by Western blotting (Fig. 5E). Furthermore, TUNEL assay of tumor tissues demonstrated that the apoptosis levels of tumor cells in ENTP5-KD group of mice were higher than those in the control group (Fig. 5F). These data suggest that knockdown of ENTPD5 inhibits the growth and promotes the apoptosis of lung cancer cells *in vivo*.

## Discussion

Lung cancer prognosis-related markers play important roles in many processes, such as tumor cell growth, apoptosis, tumor angiogenesis, and tumor cell invasion. In lung cancer, the introduction of targeted agents in patients who carry genetic abnormalities has resulted in better clinical outcomes with better quality of life. Genomic instability or selection leads to aberrations that can be grouped into six essential pathways: i) the acquisition of self-sufficient or autonomous growth signals; ii) insensitivity to growth-inhibitory signals; iii) resistance to signals of apoptosis; iv) unlimited proliferation potential; v) sustained angiogenesis; and vi) invasion and metastasis [17]. Cancer cells manifest complex genetic aberrations that occur during multi-stage carcinogenesis. Some molecular aberrations are more likely than others to influence the clinical behavior of cancer, including the risk of metastasis. Such aberrations, once identified, could potentially serve as prognostic markers, which are tumor characteristics that may influence and predict the clinical outcome of cancer patients.

The overexpression of some markers, such as vascular endothelial growth factor [18], epidermal growth factor receptor [4–6], human epidermal growth factor receptor-2 [7], p53 [8] and B-cell lymphoma-2 [9], has shown correlation with poor prognosis. In this study, immunohistochemistry analysis confirmed the specific overexpression of ENTPD5 protein in lung cancer tissues. Further investigations on overall survival suggest that enhanced expression of ENTPD5 is associated with poorer prognosis. These observations reveal the potential of ENTPD5 as a prognostic indicator.

ENTPD5 is identified as a key component in the Akt/phosphatidylinositol 3-kinase/phosphatase and tensin homolog regulatory loop, capable of synergizing aerobic glycolysis [19,20]. Some researchers show that overexpression of ENTPD5 affects the growth of many tumors, such as gliomablastoma [20], breast cancer [21], cervical cancer [22], testicular germ cell tumor [23], and laryngeal neoplasia [24]. Tumorigenesis is a multi-step process involving cell growth, invasion, metastasis, and angiogenesis. Investigation into the effect of ENTPD5 in cellular progression process shows that down-expression of ENTPD5 in A549 and PC9 cell lines significantly decreases the degrees of cell proliferation, apoptosis and migration. Collectively, these data indicate that ENTPD5 might act as an oncogene to play important roles in regulating various cellular processes that promote neoplastic progression at multiple levels. Nevertheless, the mechanisms by which ENTPD5 exerts its effects in specific signaling pathways need further studies.

NSCLC cells are apoptosis-resistant. Strategies to de-repress endogenous caspase inhibitors in NSCLC suggest promising activity in model systems and present a rational and entirely novel strategy for improving treatment of lung cancer. Apoptosis is orchestrated by the sequential activation of caspases, a family of cysteine proteases with specificity for aspartic acid residues. Two pathways may mediate apoptosis: i) the death receptors pathway (tumour necrosis factor, Fas ligand or TRAIL receptors) that activates caspase 8 and 10, and ii) the mitochondrial pathway that activates caspase 9. Both pathways lead to effector caspases 3, 6 and 7 [25]. Our research has confirmed the relationship between ENTPD5 and Caspase 3 in cellular level and animal experiments. Understanding the molecular basis of this phenotype is critical, if therapy is to move beyond the therapeutic plateau that has been reached with conventional chemotherapy. Caspase 3 expression may be associated with sensitive process of chemotherapy or radiation [26]. MacCarthy et al. reported that abnormal expression of ENTPD5 in human colorectal carcinoma cells could influence ATP levels and the resistance to oxaliplatin, a colorectal cancer-relevant chemotherapeutic agent [27]. Villar et al. also found the relationship between ENTPD 5 and chemotherapy response in prostate cancer 15]. It seems that ENTPD5 expression may not only be a powerful reporter for the metabolic shift in tumor cells, but also a possible predictor for tumor chemotherapy responses. This study found that ENTPD5 inhibited the apoptosis of lung cancer cells through caspase 3. However, the relationship between chemotherapy effect and ENTPD5 needs further research in the future. In conclusion, ENTPD5 gene is crucial in NSCLC. It could be potentially used to monitor prognosis or to guide appropriate therapeutic regimens.
